# Leukocyte Telomere Length in Young Adults Born Preterm: Support for Accelerated Biological Ageing

**DOI:** 10.1371/journal.pone.0143951

**Published:** 2015-11-30

**Authors:** Carolina C. J. Smeets, Veryan Codd, Nilesh J. Samani, Anita C. S. Hokken-Koelega

**Affiliations:** 1 Department of Pediatrics, subdivision of Endocrinology, Erasmus University Medical Center, Rotterdam, The Netherlands; 2 Dutch Growth Research Foundation, Rotterdam, The Netherlands; 3 Department of Cardiovascular Sciences, University of Leicester, Leicester, United Kingdom; 4 NIHR Leicester Cardiovascular Biomedical Research Unit, Glenfield Hospital, Leicester, United Kingdom; University of Newcastle, UNITED KINGDOM

## Abstract

**Background:**

Subjects born preterm have an increased risk for age-associated diseases, such as cardiovascular disease in later life, but the underlying causes are largely unknown. Shorter leukocyte telomere length (LTL), a marker of biological age, is associated with increased risk of cardiovascular disease.

**Objectives:**

To compare LTL between subjects born preterm and at term and to assess if LTL is associated with other putative cardiovascular risk factors at young adult age.

**Methods:**

We measured mean LTL in 470 young adults. LTL was measured using a quantitative PCR assay and expressed as T/S ratio. We analyzed the influence of gestational age on LTL and compared LTL between subjects born preterm (n = 186) and at term (n = 284). Additionally, we analyzed the correlation between LTL and potential risk factors of cardiovascular disease.

**Results:**

Gestational age was positively associated with LTL (*r* = 0.11, p = 0.02). Subjects born preterm had shorter LTL (mean (SD) T/S ratio = 3.12 (0.44)) than subjects born at term (mean (SD) T/S ratio = 3.25 (0.46)), p = 0.003). The difference remained significant after adjustment for gender and size at birth (p = 0.001). There was no association of LTL with any one of the putative risk factors analyzed.

**Conclusions:**

Young adults born preterm have shorter LTL than young adults born at term. Although we found no correlation between LTL and risk for CVD at this young adult age, this biological ageing indicator may contribute to CVD and other adult onset diseases at a later age in those born preterm.

## Introduction

Nowadays, 5–13% of all newborns in developed countries are born preterm (i.e. gestational age <37 weeks) [1]. Next to respiratory morbidity and neurodevelopmental impairment, the ex-preterm infant phenotype is also characterized by adverse metabolic health in later life [2]: Already at a young adult age, ex-preterms have a higher risk for age-associated diseases, such as cardiovascular disease (CVD) [3–7] and have increased cardiovascular mortality [8]. The fetal origin hypothesis [9] states that this increased risk is programmed during fetal life and that adverse events during pregnancy lead to reprogramming, causing diseases in later adulthood. This way, subjects born preterm, who have a low birth weight and more stress during early life, could be programmed to a different health outcome in later life [10], but the exact underlying mechanism is unknown. Because risk for CVD is age-associated, we hypothesized that accelerated ageing might be one of the mechanisms linking preterm birth and higher risk for CVD.

Telomeres are noncoding repetitive DNA sequences at the end of each chromosome. Their primary function is to maintain genomic stability [11,12]. Due to the inability of DNA polymerase to fully replicate the end of the chromosome, telomeres shorten with each cell division. When telomeres are reduced to a critical length, the cell enters a state of arrest (i.e. cell senescence) [13,14]. In population studies, telomere length declines with increasing age, which makes leukocyte telomere length (LTL) a usable index for biological ageing [13]. Also, several studies found correlations between shorter LTL and age-associated diseases [15–18].

Studies reporting on the relation between telomere length and preterm birth are scarce and contradictive. A small study (n = 26) measured LTL in umbilical cords of neonates and found no difference between LTL in preterm and full-term controls [19]. Henckel et al. [20] found shorter telomere length in ex-preterms with bronchopulmonary dysplasia at the age of 10 years, suggesting a faster telomere attrition rate in these children. Two studies measuring telomere length at an adult age found no differences between ex-preterms and controls [21,22]. Because of the large methodologic differences between these studies (i.e. salivary vs. leukocyte telomere length; neonates vs. children vs. adults, TRF vs. PCR-based measurements) many gaps remain in the current literature on the relation between preterm birth and telomere length. This was also emphasized by a recent review on telomere length and preterm birth [2].

We hypothesized that accelerated biological ageing may at least partly explain the increased risk of CVD in subjects born preterm and used LTL as an ageing biomarker to test this hypothesis. We therefore investigated the correlation between gestational age and LTL and analyzed differences in LTL between subjects born preterm and at term. Additionally, we assessed if, at the age of 21 years, putative risk factors for later CVD (i.e. body composition, blood pressure, lipid levels, insulin sensitivity and the inflammatory biomarker high sensitivity C-reactive protein) correlate with shorter LTL.

## Materials and Methods

### Subjects

The study population consisted of 470 healthy individuals, aged 18–24 years [23,24]. Subjects born preterm (gestational age <37 weeks, n = 186) had been admitted to the neonatal intensive care unit of the Erasmus University Medical Centre shortly after birth. Subjects born at term were randomly selected from hospitals and schools in The Netherlands. All participants fulfilled the same inclusion criteria: 1) age 18–24 yr; 2) born singleton; 3) Caucasian; 4) uncomplicated neonatal period without signs of severe asphyxia (defined as an Apgar score <3 after 5 min), without sepsis or long-term complications of respiratory ventilation and/or oxygen supply of 2 week during the neonatal period. Subjects were excluded if they had been suffering from any serious complication or condition (including necrotizing enterocolitis, intraventricular hemorrhage with a degree of three or more, spastic hemiplegia, or quadriplegia), from any disease or if they had an endocrine or metabolic disorder, chromosomal defect, syndrome, or serious dysmorphic symptoms suggestive for a yet unknown syndrome.

The Medical Ethics Committee of Erasmus Medical Centre approved the study. Written informed consent was obtained from all participants.

### Measurements

Participants were invited to visit Erasmus University Medical Centre. Prior to the visit, participants fasted for at least 12 hours and abstained from smoking and alcohol for at least 16 hours.

Birth data regarding gestational age and birth size were obtained from hospital records, primary health care records and general practitioner records. Information regarding socioeconomic status (SES) and smoking of the participants was obtained using questionnaires. Education level of the participants was used as socioeconomic indicator to determine SES [25]. Height was measured to the nearest 0.1 cm (Harpenden stadiometer), weight to the nearest 0.1 kg (Servo Balance KA-20-150S). Lean body mass and fat mass were measured on one Dual-energy X-ray Absorptiometry (DXA) machine (Lunar Prodigy, GE Healthcare, Chalfont St Giles, England). Systolic and diastolic blood pressure (SBP and DBP) were measured after 10 minutes at rest, in the sitting position, using the non-dominant arm with an automatic device (Accutorr Plus, Datascope Corp, Montvale, New Jersey) [26] every five minutes for one hour and the mean value was taken to reflect the resting blood pressure. To measure insulin sensitivity (Si), which plays an important role in the pathogenesis of Diabetes Mellitus type 2 (DM2), a frequent sampled intravenous glucose tolerance (FSIGT) test with Tolbutamide was performed [27]. Si quantifies the capacity of insulin to promote glucose disposal and was calculated using Bergman’s minimal model (MINMOD 6.01, copyright R.N. Bergman).

### Laboratory Methods

After centrifugation, all blood samples were kept frozen until assayed (-80°C). For measurement of hsCRP, an important predictor of future atherosclerotic events, an in-house-high-sensitivity ELISA with polyclonal rat CRP antibodies for catching and tagging (DAKO, Denmark) was used. Total cholesterol level was measured using the CHOD-PAP and the GPO-PAP reagent kit (Roche Diagnostics, Mannheim, Germany). High-density lipoprotein (HDL) cholesterol level was measured using a homogeneous enzymatic colorimetric assay (Roche Diagnostics). Low-density lipoprotein (LDL) cholesterol was calculated using the Friedewald formula: LDL cholesterol level in mmol/L = total cholesterol–HDL cholesterol level– 0.45 x level of triglycerides.

### LTL assessment

Genomic DNA was isolated from peripheral leukocytes using standard procedures. All LTL measurements were made in the same laboratory at the University of Leicester, without knowledge of birth status. Mean LTL was measured by the quantitative PCR-based technique as previously described [28,29]. Telomere sequence copy number (T) was compared with a single copy gene number in the genome 36B4 (S) and telomere length expressed as a T/S ratio. All T and S values were calculated relative to a calibrator DNA (genomic DNA from the K562 cell line) that was included on every plate. This allows correction for inter-run variation. For quality control, all samples were checked for concordance between duplicate values. Samples showing a difference of greater than 0.2 cycles in the take-off value or amplifying outside of the linear range of the assay were excluded and re-run. Reproducibility of the assay was tested by re-running samples on separate days. The mean inter-run CV for the T/S ratio was 3.13%.

### Statistical analysis

Standard deviation (SD)-scores for birth length and birth weight were calculated in order to correct for gestational age and gender [30]. SD-scores for adult height and weight were calculated to correct for gender and age [31]. SD-scores for blood pressure, fat mass percentage and lean body mass were calculated to correct for gender and height. SD-scores were calculated using growth analyser software (http://www.growthanalyser.org).

Baseline characteristics of normally distributed data are presented as mean (SD) and of non-normally distributed data as median (interquartile range (IQR)). ANOVA (continuous data) and Chi square tests (categorical data) were used to determine differences between participants born either preterm or at term. The association between gestational age and LTL was determined using multiple linear regression analysis. After assessing the linear correlation (Model A), adjustments were made for age, gender, birth length SDS, birth weight SDS and adult height SDS (Model B). The interaction term birth length SDS * adult height SDS was added to the analysis because the study group had been selected on birth length and adult height, in order to ensure that the effect of these variables was modeled correctly. Additionally, we adjusted for smoking and SES (Model C). Difference in LTL between subjects born preterm and at term was analyzed with an independent samples t-test. In an ANCOVA model, we additionally corrected for gender, birth length SDS and birth weight SDS. The association between LTL and risk factors of CVD was assessed with linear regression, with LTL as dependent variable and the different risk factors as independent variables. Finally, we assessed if there were differences in these risk factors between subjects in the bottom quartile for LTL and subjects in the upper quartile for LTL, using independent samples t-tests.

Results were considered statistically significant if the p-value was <0.05. Statistical package SPSS version 21.0 (SPSS, Inc., Chicago, IL) was used for all analyses.

## Results

Characteristics of the study population are shown in Table 1. The total population consisted of 470 subjects with a mean (SD) T/S of 3.20 (0.46). Gestational age varied between 27 and 43 weeks, with 186 subjects born preterm (gestational age <37 weeks) and 284 subjects born at term (gestational age >37 weeks). Birth weight SDS, adult height SDS and weight SDS were significantly higher in subjects born preterm. Diastolic blood pressure SDS and total cholesterol were significantly lower in subjects born preterm.

**Table 1:** Clinical characteristics and risk factors for CVD of the total study population and the subjects born preterm and at term.

		Total group(n = 470)	Preterm(n = 186)	At Term(n = 284)	p-value
Male/Female		204/266	90/96	114/170	0.08
Age (yrs)^1^		20.9 (1.7)	20.9 (1.7)	20.9 (1.7)	0.61
Gestational age (wks)^1^		36.7 (3.9)	32.5 (2.4)	39.4 (1.5)	**<0.001**
Birth weight (SDS)^1^		-0.97 (1.6)	-0.57 (1.9)	-1.22 (1.3)	**<0.001**
Birth length (SDS)^1^		-1.44 (1.6)	-1.36 (1.9)	-1.49 (1.4)	0.41
Height SDS^1^		-0.85 (1.3)	-0.39 (1.0)	-1.13 (1.3)	**<0.001**
Weight SDS^1^		-0.48 (1.3)	-0.23 (1.2)	-0.66 (1.4)	**0.001**
BMI SDS^1^		0.00 (1.2)	0.01 (1.2)	-0.00 (1.1)	0.94
Fat mass % SDS^1^		0.00 (1.0)	0.30 (1.7)	-0.02 (0.9)	0.19
Lean body mass SDS^1^		-0.48 (1.0)	-0.42 (1.1)	-0.49 (1.0)	0.76
SBP SDS^1^		-0.08 (0.7)	-0.03 (0.7)	-0.12 (0.8)	0.24
DBP SDS^1^		0.21 (0.5)	0.12 (0.5)	0.29 (0.5)	**0.002**
TC (mmol/L)^1^		4.4 (0.9)	4.3 (0.8)	4.5 (1.0)	**0.005**
HDLc (mmol/L)^1^		1.4 (0.4)	1.4 (0.3)	1.4 (0.4)	0.49
LDLc (mmol/L)^1^		2.6 (0.8)	2.5 (0.7)	2.7 (0.9)	0.12
Tg (mmol/L)^1^		1.0 (0.5)	1.0 (0.5)	1.0 (0.5)	0.47
hsCRP (mg/l)^2^		1.12 (3.1)	1.24 (3.7)	0.75 (1.6)	0.78
Si * 10^−4^/min (μU/ml)^2^		6.32 (6.2)	6.41 (6.4)	5.59 (6.7)	0.13
Smoking (%)		26.8	26.7	27.9	0.53
SES (%)	1	12.4	14.1	11.5	
	2	26.6	30.2	24.5	0.70
	3	60.9	55.7	64.0	

^1^Values are given as mean (sd).

^2^Values are given as median (IQR).

p-values below 0.05 are shown in bold type. Preterm = gestational age <37 wks, at term = gestational age >37 wks. BMI = Body mass index; DBP = diastolic blood pressure; HDLc = high-density lipoprotein cholesterol; hsCRP = C-reactive protein; LDLc = low-density lipoprotein cholesterol; SBP = systolic blood pressure; SES = Socioeconomic status; Si = Insulin sensitivity; TC = Total cholesterol; Tg = triglycerides.

### Variables at birth and at young adult age influencing telomere length

We evaluated the relative contribution of several variables at birth and at young adult age to LTL in a multiple regression analysis (Table 2). There was a significant positive correlation between gestational age and LTL (Model A, R^2^ = 0.01, p = 0.02). Adding age, gender, height SDS, birth weight SDS, birth length and the interaction term birth length SDS * adult height SDS to the model (Model B, R^2^ = 0.04), improved the model and the correlation between gestational age and LTL remained significant (p = 0.03). Also, a positive association was found between female gender and LTL (p = 0.002). Gestational age and gender both remained significant after adding smoking and socioeconomic status (Model C, R^2^ = 0.05) to the analysis (p = 0.04 and p = 0.003, respectively).

**Table 2:** Multiple regression for variables influencing telomere length in the total study population.

	Model A		Model B		Model C	
Variables	ß	p	ß	p	ß	p
GA (wks)	0.01	**0.02**	0.02	**0.03**	0.02	**0.04**
Age (yrs)			0.02	0.30	0.01	0.46
Female gender			0.16	**0.002**	0.16	**0.003**
Adult Height SDS			-0.02	0.48	-0.03	0.27
Birth weight SDS			0.01	0.64	0.00	0.95
Birth length SDS			0.01	0.81	0.02	0.50
BL*AH (SDS)			0.00	0.86	0.01	0.56
Smoking					-0.07	0.26
SES					-0.01	0.83
Overall p-value	0.02		0.006		0.008	
R^2^ adjusted	0.01		0.04		0.05	

p-values below 0.05 are shown in bold type. GA = Gestational age; BL*AH = Interaction term birth length * adult height. SES = Socioeconomic status (Low and middle socioeconomic status are used as reference for SES analyses).

### Telomere length in subjects born preterm versus at term

**Fig 1:**
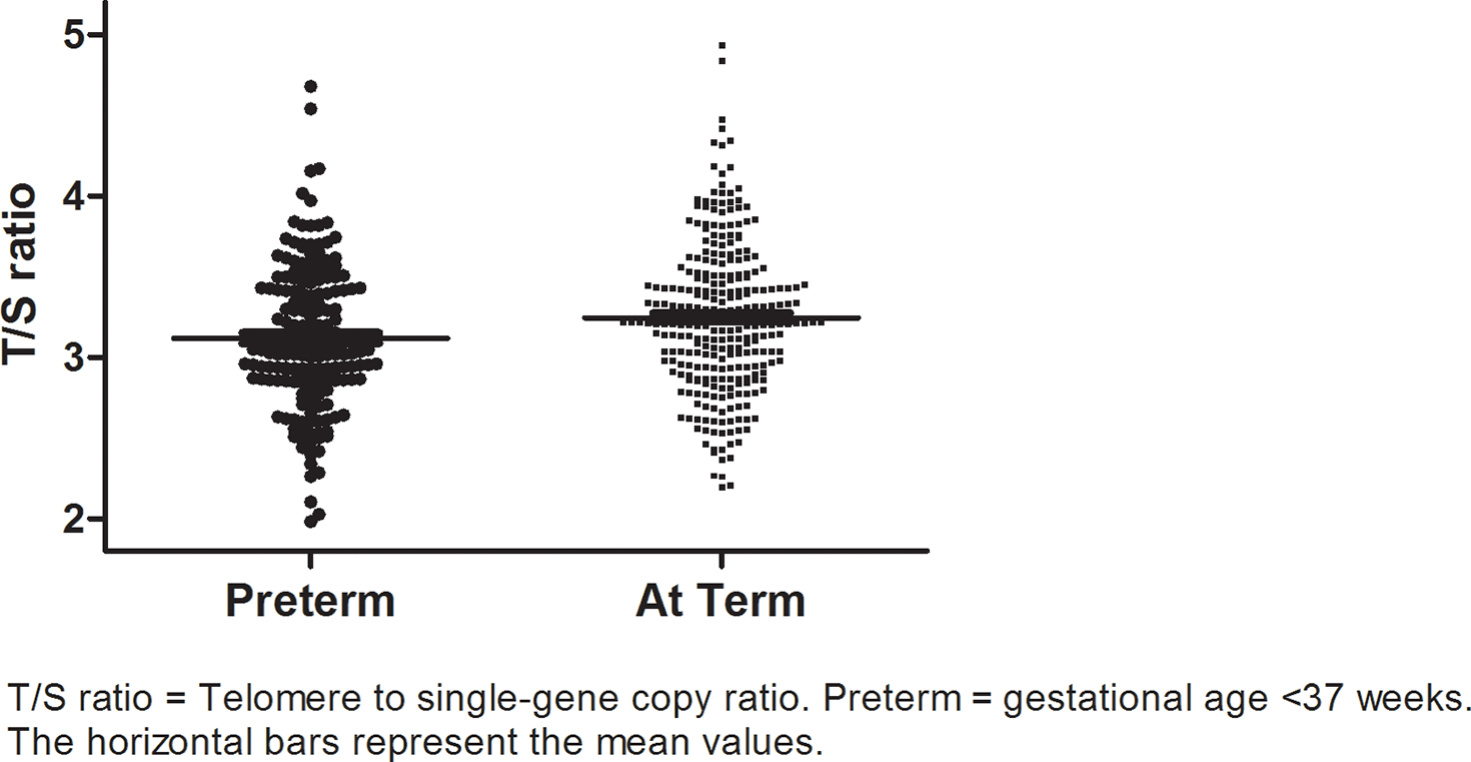
Distributions of mean telomere lengths in subjects born preterm and at term. T/S ratio = Telomere to single-gene copy ratio; Preterm = gestational age < 37 wks; The horizontal bars represent the mean values.

Fig 1 shows the difference in LTL between subjects born preterm and at term. Unadjusted for possible confounders, subjects born preterm had significantly shorter LTL than subjects born at term (mean (SD) T/S of 3.12 (0.44) and 3.25 (0.46), respectively, p = 0.003). This difference remained significant after correction for gender, birth length SDS and birth weight SDS (Table 3, p = 0.001).

**Table 3:** Relation of telomere length with preterm birth and cardiovascular risk factors.

	Telomere length	
	ß	p
Preterm	-0.13	**0.003**
Preterm adjusted^1^	-0.15	**0.001**
SBP SDS	-0.01	0.79
DBP SDS	-0.00	0.99
Fat mass % SDS	0.02	0.58
Lean body mass SDS	-0.04	0.13
TC (mmol/l)	0.04	0.09
LDLc (mmol/l)	0.04	0.26
HDLc (mmol/l)	-0.01	0.90
Tg (mmol/l)	0.05	0.31
hsCRP (mg/l)	0.00	0.95
Si * 10^−4^/min (μU/ml)	0.00	0.54

p-values below 0.05 are shown in bold type. ß coefficients equate to the difference in telomere length per unit change in the variable. DBP = diastolic blood pressure; HDLc = high-density lipoprotein cholesterol; hsCRP = C-reactive protein; LDLc = low-density lipoprotein cholesterol; SBP = systolic blood pressure; Si = Insulin sensitivity; TC = Total cholesterol; Tg = triglycerides. The variable preterm equates to the difference between subjects born preterm and the reference group subjects born at term.

^1^ = Adjusted for gender, birth weight SDS and birth length SDS.

### Relationship between telomere length and CVD and DM2 risk factors

We assessed the relation between LTL and several putative CVD risk factors, including insulin sensitivity (Si), at 21 years of age (Table 3). There were no significant associations between LTL and these variables. Moreover, these risk factors did not significantly differ between subjects in the bottom and upper quartile for LTL (S1 Table).

## Discussion

In this study, we analyzed the association between gestational age and LTL in 470 young adults and compared LTL between subjects born preterm and at term. We found that gestational age is positively associated with LTL and that subjects born preterm have shorter LTL than subjects born at term. This difference remained significant after correction for birth length and birth weight, indicating an independent effect of gestational age on LTL, not confounded by birth size. We also found that females had longer telomeres than males, which is in concordance with earlier studies [29]. At this young age, no relation between LTL and other putative risk factors for CVD was found.

Thus far, there is limited literature on the association between preterm birth and LTL and the results are contradictive. Friedrich et al. [19] measured telomere length in umbilical cords of neonates and found no relation between preterm birth and telomere length. Although this study provides valuable insights on telomere length at birth, it lacks data on telomere length at a later age and had a very small sample size (n = 26). Henckel et al. [20] measured telomere length in children at the age of 10 and found that ex-preterm children with bronchopulmonary dysplasia had shorter telomeres than at term children with asthma, suggesting a faster telomere attrition in preterm infants, already present at the age of 10 years old. However, these results are difficult to compare to ours as children with asthma or bronchopulmonary dysplasia comprise a very different study population. A very recent study of Hadchouel et al. [21] found a correlation between telomere length and abnormal airflow in adolescents born extremely preterm. However, there was no association found between telomere length and gestational age or perinatal events, suggesting that preterm birth *per se* is not a risk factor for shortening of telomeres. In contrast to our study, telomere length was measured in saliva in that study, which questions the comparability of their results to our findings. Kajantie et al. [22] described the relation between several birth factors and adult LTL. In contrast to our study, no correlation was found between preterm birth and LTL. One of the reasons for the different results could be that the percentage of preterms was very low compared to those born at term (5.9%).

Previous studies have highlighted oxidative stress as an important determinant of LTL [32,33] and showed that intrauterine stress causes shorter LTL [34–36]. Since pregnancies resulting in preterm birth are often accompanied by increased stress exposure [37] and preterm born infants are frequently exposed to stressful events, we think it is plausible that oxidative stress is one of the explanations for the difference in LTL between those born preterm and at term. Other determinants of LTL are replicative stress and genetic factors [38]. Most preterm born infants go through a phase of slow postnatal growth due to feeding problems, followed by a phase of accelerated growth (i.e. catch-up growth) mostly from term age onwards. Since catch-up growth can induce replicative stress, preterms could be exposed to increased replicative stress, causing shorter telomeres. To analyze this, we we added both birth length SDS and adult height SDS in the multiple regression analysis which stands for the change in height SDS during childhood. If catch-up growth influences LTL, we would have expected a significant association between the change in height SDS during childhood and LTL. Because we did not find this, we think that replicative stress does not explain the findings in our study. This is in concordance with previous studies [22,39]. To our knowledge, there is no reason to believe that parents of preterm infants have shorter LTL than those of term infants and we thus do not consider genetic factors to be the cause for the difference in LTL between preterms and terms. A future study that measures LTL and oxidative stress biomarkers during fetal and early postnatal life and, subsequently at a later age, would be a good way to evaluate if increased perinatal oxidative stress is indeed the mechanism behind shorter LTL in those born preterm. Ideally, LTL would be measured in parents too, to analyze the influence of genetic factors.

To provide a more meaningful context in terms of kilobases for the observed difference in T/S ratio between those born preterm and those born at term, we used data from a previous study from the same laboratory where a comparison had been made between LTL measured by PCR and in kilobases by Southern blotting [40,41]. On this basis, a difference in T/S ratio of 0.13 equates to approximately 180 base pairs. Since age-related decline in LTL has been reported to be between 15 and 35 base pairs per year [15,18,41,42] the difference of 180 base pairs equates to approximately 5 to 12 years. This might suggest that young adults born preterm are 5–12 biological years older than young adults born at term with a comparable calendar age. However, since this conversion is based on data of a previous study, we have to be cautious with drawing definitive conclusions from this calculation. As longitudinal telomere length measurements were lacking and because all participants had the same age, we were unable to calculate the mean telomere attrition rate/year in our cohort. Therefore we cannot take inter-individual telomere attrition rates into account. Previous studies showed that telomere attrition rates vary at different ages, with the most rapid loss early in life, followed by a plateau between age 3–4 and young adulthood, and gradual attrition later in life [43–45]. Since the participants of our study had a comparable calendar age, the conversion from base pairs to years was not influenced by this.

Previously, we have shown that several putative risk factors for CVD are already increased at a young age in subjects born preterm [3,4]. We therefore investigated whether there was an association between LTL and these risk factors. However, in these young adults, correlations between LTL and these risk factors for CVD were not found. This is in concordance with earlier studies, indicating that the association between LTL and CVD is independent of risk factors for CVD, including markers of inflammation [15,40,41]. Although we found no correlation between LTL and risk for CVD at this young adult age, we think that this biological ageing indicator may contribute to CVD and other adult onset diseases at a later age in those born preterm. It would therefore be very interesting to analyze how LTL and CVD progress over time when these young adults reach their 30s and 40s.

In conclusion, our data show that gestational age is positively correlated with LTL and that young adults born preterm have shorter LTL than young adults born at term. This could reflect pre- and postnatal oxidative stress and in turn could partly explain the association between preterm birth and later life risk of CVD. Since the prevalence of preterm birth and survival is rapidly increasing, our results are of clinical relevance for a large and increasing number of subjects worldwide.

## Supporting Information

S1 Table Difference in cardiovascular risk factors between highest and lowest quartile telomere length. Values are given as mean (sd). DBP = diastolic blood pressure; HDLc = high-density lipoprotein cholesterol; hsCRP = high sensitivity C-reactive protein; LDLc = low-density lipoprotein cholesterol; SBP = systolic blood pressure; Si = insulin sensitivity; TC = total cholesterol; Tg = triglycerides.
(PDF)
