# Supplementary material for: Leukocyte Telomere Length in Young Adults Born Preterm: Support for Accelerated Biological Ageing
Source: PLoS One. 2015 Nov 30;10(11):e0143951. doi: 10.1371/journal.pone.0143951 (PMC4664383; doi:10.1371/journal.pone.0143951)
Supplement: S1 Table — Values are given as mean (sd). DBP = diastolic blood pressure; HDLc = high-density lipoprotein cholesterol; hsCRP = high sensitivity C-reactive protein; LDLc = low-density lipoprotein cholesterol; SBP = systolic blood pressure; Si = insulin sensitivity; TC = total cholesterol; Tg = triglycerides. (PDF) [file pone.0143951.s001.pdf]

Supplement Table. Difference in cardiovascular risk factors between highest and lowest quartile telomere length

	**Lowest quartile LTL**	**Highest quartile LTL**	**p-value**
SBP SDS	-0.02 (0.7)	-0.08 (0.8)	0.58
DBP SDS	0.20 (0.5)	0.21 (0.6)	0.93
Fat mass % SDS	0.10 (1.1)	0.19 (1.1)	0.63
Lean body mass SDS	-0.61 (1.0)	-0.82 (0.9)	0.17
TC (mmol/l)	4.38 (0.9)	4.57 (0.9)	0.12
LDLc (mmol/l)	2.55 (0.7)	2.64 (0.7)	0.37
HDLc (mmol/l)	1.38 (0.4)	1.38 (0.4)	0.96
Tg (mmol/l)	0.99 (0.6)	1.06 (0.5)	0.33
hsCRP (mg/l)	5.11 (7.4)	4.43 (7.2)	0.53
Si * 10^{-4}/min (µU/ml)	6.72 (3.8)	7.72 (6.1)	0.28

Values are given as mean (sd).

DBP = diastolic blood pressure; HDLc =high-density lipoprotein cholesterol; hsCRP = C-reactive protein; LDLc = low-density lipoprotein cholesterol; SBP = systolic blood pressure; Si = Insulin sensitivity; TC = Total cholesterol; Tg = triglycerides.
